# Characteristics of acute ischemic stroke in hospitalized patients in Tibet: a retrospective comparative study

**DOI:** 10.1186/s12883-020-01957-0

**Published:** 2020-10-21

**Authors:** Yuxuan Lu, Cidan Zhuoga, Haiqiang Jin, Feiqi Zhu, Yuhua Zhao, Zhijie Ding, Shihua He, Ailian Du, Jun Xu, Jingjing Luo, Yongan Sun

**Affiliations:** 1 Department of Neurology, Peking University First Hospital, No. 8, Xishiku St, Xicheng District, Beijing, 100034 China; 2 Department of Neurology, People’s Hospital of Tibet Autonomous Region, Lhasa, 850000 Tibet China; 3 Cognitive Impairment Ward of Neurology Department, The Third Affiliated Hospital of Shenzhen University Medical College, Shenzhen, 518005 China; 4 Department of Neurology, Tongren Hospital, Shanghai Jiaotong University School of Medicine, Shanghai, 200050 China; 5 Department of Cognitive Neurology, China National Clinical Research Center for Neurological Diseases (NCRC-ND), Beijing Tiantan Hospital, Capital Medical University, Beijing, 100070 China

**Keywords:** High altitude, Acute ischemic stroke, Risk factor, Stroke subtype, Young adult stroke

## Abstract

**Background:**

Numerous studies on acute ischemic stroke (AIS) have been conducted at low-altitude regions, and the related findings have been used to guide clinical management. However, corresponding studies at high altitude are few. This study aimed to analyse the clinical characteristics of AIS patients at high-altitude regions through a hospital-based comparative study between Tibet and Beijing.

**Methods:**

This study included the diagnoses of AIS patients from People’s Hospital of Tibet Autonomous Region (PHOTAR) and Peking University First Hospital (PUFH) between 1 January 2014 and 31 December 2017, where data including patient demographics, treatment time, onset season, risk factors, infarction location, laboratory data, image examination results, treatments, and AIS subtype were collected and compared. Continuous and categorical variables were analysed with a two-sample t-test or Wilcoxon rank sum test and chi-square test, respectively. Significant risk factors were examined with binary logistic regression analysis.

**Results:**

In total, 236 and 1021 inpatients from PHOTAR and PUFH were included, respectively. The PHOTAR patients were younger than the PUFH patients (*P* < 0.001). Young adult stroke, erythrocytosis, and hyperhomocysteinemia were more frequent in PHOTAR patients (all *P* < 0.001). Other vascular risk factors, including hypertension, diabetes mellitus, hyperlipidaemia, smoking and alcohol consumption history, were less prevalent in PHOTAR patients than in PUFH patients. The rate of intravenous thrombolysis and the rate of within intravenous thrombolysis window time were also lower in PHOTAR patients (both *P* < 0.001). The PHOTAR group also tended to have anterior circulation infarction. Erythrocytosis and hyperhomocysteinemia were independent risk factors in PHOTAR, and young adults accounted for a larger proportion of stroke cases.

**Conclusion:**

In Tibet, AIS patients were relatively younger, and anterior circulation infarctions were more common. Erythrocytosis and hyperhomocysteinemia may contribute to these differences. Here, young adult stroke also accounted for a higher proportion, and this may be associated with erythrocytosis. Our findings present the first hospital-based comparative study in Tibet and may contribute to policies for stroke prevention in this region.

## Background

Stroke is a leading cause of mortality and disability worldwide, accounting for about 5% of all disability-adjusted life years and 10% of all deaths [1]. In China, ischemic stroke (IS) contributes 79.1% of all stroke cases nationwide [2–4]. There are numerous studies on ischemic stroke at low-altitude regions, while relevant studies at high altitude are few. Tibet has unique geographical features with high altitudes and extreme atmospheric conditions; the clinical characteristics of acute ischemic stroke (AIS) in Tibet may differ from that at lower heights due to chronic hypoxia and high haemoglobin (HGB). Previous studies have yielded controversial findings related to the association between high altitude, the incidence of stroke, and the role of HGB [5, 6]. Additionally, the incidence of stroke in young adults is higher in Tibet than in other areas of China, and the primary type of stroke is ischemic stroke [6, 7]. Furthermore, there are very few studies on AIS risk factors and AIS subtypes. This study aimed to provide a profile of AIS in Tibet and high-altitude regions by conducting a comparative study of hospitalized patients from two parts of China with different elevations.

## Methods

### Hospital and subjects

The People’s Hospital of Tibet Autonomous Region (PHOTAR) in Tibet and Peking University First Hospital (PUFH) in Beijing were chosen as the comparative hospitals. This study included consecutive patients with a principal diagnosis of AIS at discharge who were admitted to one of the above two facilities between 1 January 2014 and 31 December 2017. The diagnosis was based on the International Classification of Diseases 10th Revision, and all participants were examined using magnetic resonance imaging (MRI) to confirm new infarcts. Patients without new infarcts were excluded. Data, including patient demographics, treatment time, risk factors, infarction location, laboratory data, image examination results, treatments, and AIS subtype, were collected in a unified form.

### Definitions and data processing

Young adult stroke was defined as stroke with an onset age of less than 45 years. Intravenous thrombolysis window time (IVTWT) was referred to as the 6 h-period from symptom onset to thrombolysis [8, 9]. Hypertension was specified as systolic and/or diastolic blood pressure of > 140 mmHg and > 90 mmHg, respectively, on more than two distinct occasions or history of antihypertensive treatment. Diabetes mellitus (DM) was determined as a confirmed diagnosis of type 1 or 2 DM, or glycosylated HGB of ≥6.5% at the time of admission. Hyperlipidaemia was designated as low-density lipoprotein (LDL) cholesterol level of ≥2.6 mmol/L at the time of admission or a history of hyperlipidaemia or lipid-lowering treatment. Stroke history included previous strokes confirmed by neurologists and supported by imaging findings. Atrial fibrillation (AF) covered any history or manifestations of AF, as identified by an electrocardiogram during hospitalisation. Erythrocytosis was stipulated as HGB level ≥ 185 g/L and ≥ 165 g/L for males and females, respectively, at the time of admission or a history of erythrocytosis. Hyperhomocysteinemia was interpreted as homocysteine levels ≥15 μmol/L or a history of treatment by related drugs. The severity of carotid artery atherosclerosis was calculated according to carotid artery ultrasound results and classified in the ascending order by hazard level as follows: normal, intima thickening, plaque, arteriostenosis, and arterial occlusion. Intima thickening was defined as intima-media thickness (IMT) > 1 mm; plaque was defined as IMT > 1.5 mm; arteriostenosis was defined as a reduction of the inner-diameter by > 50%; and occlusion was defined as no indication of blood flow. Infarction locations, confirmed by anatomical MRI, were classified into the basal ganglion, cerebral lobe, brainstem, thalamus, cerebellum, and/or corona radiata regions; supratentorial location included the basal ganglion, cerebral lobe, thalamus and corona radiata regions; and deep location included the basal ganglion and thalamus. When several positions were affected, all were taken into consideration.

### AIS classification

The Trial of Org 10,172 in Acute Stroke Treatment (TOAST) and the Oxfordshire Community Stroke Project (OCSP) classifications were applied [10, 11]. According to the TOAST classification, AIS was divided into five subtypes: (1) large-artery atherosclerosis (LAAS), (2) cardioembolism, (3) small-vessel occlusion, (4) stroke of other determined aetiology, and (5) stroke of undetermined aetiology. In contrast, according to the OCSP classification, AIS was divided into four subtypes: (1) total anterior circulation infarcts (TACI), (2) partial anterior circulation infarcts (PACI), (3) posterior circulation infarcts, and (4) lacunar infarcts (LACI). Two neurologists independently categorized AIS, and any differences were resolved by involving a third neurologist.

### Treatment

The term treatment encompassed any treatments in the emergency department or during hospitalization including thrombolysis, antihypertensive drugs, antidiabetic drugs, antihyperlipidemic drugs, intracranial pressure (ICP)-lowering drugs, antiplatelet drugs, and anticoagulants. Thrombolysis was considered intravenous thrombolysis with recombinant tissue plasminogen activator (rt-PA) or urokinase, which were approved by the Chinese government [9]. ICP-lowering drugs referred to the administration of mannitol and glycerine fructose.

### Statistical analysis

Statistical analyses were performed with IBM SPSS 21.0 software package (IBM, Armonk, NY, USA). Qualitative and quantitative variables were expressed as a percentage and mean ± standard deviation (SD), respectively. Categorical variables were compared using the chi-square (χ^2^) test, while continuous variables were examined using a two-sample t-test if they were normally distributed or otherwise with Wilcoxon rank sum test. The association of ischemic stroke in Tibet with various risk factors was explored using binary logistic regression analysis. *P*-value < 0.05 was considered statistically significant.

## Results

### Demographic characteristics and risk factors of AIS patients in PHOTAR and PUFH

The sample comprised 236 (175 males, 74.2%) and 1021 AIS patients (731 males, 71.6%) from PHOTAR and PUFH, respectively. The PHOTAR patients were significantly younger than the PUFH patients (58.19 ± 14.49 years vs. 65.10 ± 13.15 years; *P* < 0.001). Young adult stroke was more predominant in PHOTAR than in PUFH (17.3% vs. 5.6%; *P* < 0.001). The rate of IVTWT differed significantly between the two hospitals, with 9.7% and 17.1% of cases within the IVTWT in the PHOTAR and PUFH groups, respectively (*P* < 0.001). Hypertension (75.6% vs. 64.8%, *P* = 0.001), DM (41.9% vs. 23.3%, *P* < 0.001), hyperlipidaemia (59.4% vs. 51.3%, *P* = 0.013), smoking history (49.7% vs. 30.1%, *P* < 0.001), alcohol consumption history (42.2% vs. 31.4%, *P* = 0.002), and stroke history (29.4% vs. 6.4%, *P* < 0.001) were significantly more common in PUFH patients than in PHOTAR patients. Erythrocytosis (28.8% vs. 0.4%, *P* < 0.001) and hyperhomocysteinemia (60.6% vs. 36.9%, *P* < 0.001) were significantly more prevalent in PHOTAR patients than in PUFH patients. In contrast, vascular stenosis was significantly more frequent in PUFH patients than in PHOTAR patients (*P* < 0.001) (Table 1).

**Table 1:** Demographic characteristics and risk factors of AIS patients in PHOTAR and PUFH

	PHOTAR (***n*** = 236)	PUFH (***n*** = 1021)	***P***-Value
**Male, n (%)**	175 (74.2)	731 (71.6)	0.43
**Average age (years)**	58.19 ± 14.49	65.10 ± 13.15	< 0.001
**Young adult stroke, n (%)**	41 (17.3)	57 (5.6)	< 0.001
**Within IVTWT, n (%)**	23 (9.7)	175 (17.1)	< 0.001
**Hypertension, n (%)**	153 (64.8)	772 (75.6)	0.001
**Diabetes mellitus, n (%)**	55 (23.3)	428 (41.9)	< 0.001
**Hyperlipidaemia, n (%)**	121 (51.3)	606 (59.4)	0.013
**Smoking history, n (%)**	71 (30.1)	507 (49.7)	< 0.001
**Alcohol consumption history, n (%)**	74 (31.4)	431 (42.2)	0.002
**Stroke history, n (%)**	15 (6.4)	300 (29.4)	< 0.001
**Atrial fibrillation, n (%)**	14 (5.9)	96 (9.4)	0.112
**Erythrocytosis, n (%)**	68 (28.8%)	4 (0.4%)	< 0.001
**Hyperhomocysteinemia, n (%)**	143 (60.6)	377 (36.9)	< 0.001
**The severity of carotid artery atherosclerosis**			<0.001
**Normal, n (%)**	104 (44.1)	41 (4.0)	
**Intima thickening, n (%)**	6 (2.5)	62 (6.1)	
**Plaque, n (%)**	117 (49.6)	626 (61.3)	
**Arteriostenosis, n (%)**	5 (2.1)	144 (14.1)	
**Arterial occlusion, n (%)**	4 (1.7)	52 (5.1)	

Data are presented as mean ± standard deviation or number (percentage)

*AIS* Acute ischemic stroke, *IVTWT* Intravenous thrombolysis window time, *PHOTAR* People’s Hospital of Tibet Autonomous Region, *PUFH* Peking University First Hospital

### Classification and location of AIS in PHOTAR and PUFH patients

While the cerebral lobes (50.0% vs. 42.4%, *P* = 0.033) and supratentorial location (83.9% vs. 73.5%, *P* < 0.001) were significantly more susceptible to infarction in PHOTAR patients than in PUFH patients, the brainstem (20.5% vs. 9.3%, *P* < 0.001), thalamus (12.2% vs. 5.9%, *P* = 0.008), corona radiata (49.4% vs. 11.9%, *P* < 0.001), and deep location (54.7% vs. 41.1%, *P* = 0.001) had higher incidences of infarction in PUFH patients than in PHOTAR patients. According to the TOAST classification, the distribution of AIS subtypes was significantly different between the two hospitals (*P* < 0.001), where the small vessel disease subtype was more prevalent in PUFH. Concerning the OSCP classification, TACI and PACI subtypes were more common in PHOTAR patients, while the LACI subtype accounted for a considerable proportion in PUFH patients (Table 2).

**Table 2:** Classification and location of AIS patients in PHOTAR and PUFH

	PHOTAR (***n*** = 236)	PUFH (***n*** = 1021)	***P***-Value
**Infarction location**			
**Basal ganglion, n (%)**	60 (25.4)	268 (26.3)	0.789
**Cerebral lobe, n (%)**	118 (50.0)	432 (42.4)	0.033
**Corona radiata, n (%)**	28 (11.9)	503 (49.4)	< 0.001
**Thalamus, n (%)**	14 (5.9)	124 (12.2)	0.008
**Cerebellum, n (%)**	20 (8.5)	79 (7.7)	0.810
**Brainstem, n (%)**	22 (9.3)	209 (20.5)	< 0.001
**Supratentorial location, n (%)**	198 (83.9)	747 (73.5)	< 0.001
**Deep location, n (%)**	74 (41.1)	360 (54.7)	0.001
**TOAST subtype**			<0.001
**LAAS, n (%)**	166 (70.3)	664 (65.0)	
**Small vessel disease, n (%)**	7 (3.0)	242 (23.7)	
**Cardioembolism, n (%)**	29 (12.3)	98 (9.6)	
**OSCP subtype**			<0.001
**TACI, n (%)**	21 (8.9)	11 (1.1)	
**PACI, n (%)**	153 (64.8)	484 (49,2)	
**POCI, n (%)**	61 (25.8)	254 (25.8)	
**LACI, n (%)**	1 (0.4)	235 (23.9)	

*AIS* Acute ischemic stroke, *LAAS* Large-artery atherosclerosis, *OSCP* Oxfordshire Stroke Classification Project, *TACI* Total anterior circulation infarcts, *PACI* Partial anterior circulation infarcts, *POCI* Posterior circulation infarcts, *LACI* Lacunar infarcts, *PHOTAR* People’s Hospital of Tibet Autonomous Region, *PUFH* Peking University First Hospital, *TOAST* Trial of Org 10,172 in Acute Stroke Treatment

### Treatment of AIS patients in PHOTAR and PUFH

Thrombolysis was performed for 0.4% and 5.2% of PHOTAR and PUFH patients, respectively (*P* = 0.001). At PHOTAR, only one person underwent thrombolytic therapy and was treated with urokinase. However, at PUFH, 53 patients underwent thrombolytic therapy, among which 37 (69.8%) were treated with rt-PA, 4 (7.5%) with urokinase, and the thrombotic agents used for 12 (22.6%) were unknown. Antihypertensives (66.7% vs. 56.4%, *P* = 0.003), antidiabetics (37.7% vs. 10.2%, *P* < 0.001), antihyperlipidemic (89.7% vs. 73.3%, *P* < 0.001), antiplatelet drugs (92.4% vs. 83.5%, *P* < 0.001), and anticoagulants (22.7% vs. 8.1%, *P* < 0.001) were more frequently used by PUFH patients, while ICP-lowering drugs (21.6% vs. 4.7%, *P* < 0.001) were more routinely used by PHOTAR patients (Table 3).

**Table 3:** Treatment of AIS patients in PHOTAR and PUFH

	PHOTAR (***n*** = 236)	PUFH (***n*** = 1021)	***P***-Value
**Thrombolysis, n (%)**	1 (0.4)	53 (5.2)	0.001
**Antihypertensive drugs, n (%)**	133 (56.4)	679 (66.7)	0.003
**Antidiabetic drugs, n (%)**	24 (10.2)	383 (37.7)	< 0.001
**Antihyperlipidemic drugs, n (%)**	173 (73.3)	914 (89.7)	< 0.001
**ICP-lowering drugs, n (%)**	51 (21.6)	48 (4.7)	< 0.001
**Antiplatelet drugs, n (%)**	197 (83.5)	941 (92.4)	< 0.001
**Anticoagulants, n (%)**	19 (8.1)	231 (22.7)	< 0.001

*AIS* Acute ischemic stroke, *PHOTAR* People’s Hospital of Tibet Autonomous Region, *PUFH* Peking University First Hospital, *ICP* Intracranial pressure

### Binary logistic regression to identify risk factors for ischemic stroke patients in Tibet

All risk factors with significant differences between two hospitals in Table 1 were analysed with binary logistic regression to explore the potential risk factors of AIS in Tibet. Here, erythrocytosis and hyperhomocysteinemia were confirmed as independent risk factors in ischemic stroke patients in Tibet (both *P* < 0.001). While coefficient of erythrocytosis indicated a relatively strong influence on patients in PHOTAR, age, DM, smoking, stroke history, and the severity of carotid artery atherosclerosis had a more pronounced effect on patients in PUFH (Table 4).

**Table 4:** Binary logistic regression to identify risk factors for ischemic stroke patients in Tibet

	Coefficient	OR	95%CI	***P***-Value
**Age, years**	−0.031	0.970	0.954–0.986	< 0.001
**Diabetes mellitus**	−0.150	0.347	0.223–0.539	< 0.001
**Smoking history**	−1.534	0.216	0.125–0.373	< 0.001
**Stroke history**	−1.465	0.231	0.113–0.473	< 0.001
**Erythrocytosis**	5.203	181.849	39.004–847.841	< 0.001
**Hyperhomocysteinemia**	0.919	2.508	1.649–3.814	< 0.001
**Severity of carotid artery atherosclerosis**	−0.548	0.578	0.490–0.682	< 0.001

*OR* Odds ratio, *CI* Confidence interval

### Comparison of risk factors for young adult stroke between PHOTAR and PUFH patients

For young adult stroke, hypertension (70.2% vs. 36.6%, *P* = 0.002), DM (47.8% vs. 12.2%, *P* = 0.001), hyperlipidaemia (64.9% vs. 41.5%, *P* = 0.036), and stroke history (17.5% vs. 2.4%, *P* = 0.023) were predominantly found in PUFH patients. And carotid artery atherosclerosis (*P* = 0.001) was more severe in PUFH patients, erythrocytosis (29.3% VS. 0%, *P* < 0.001) was more frequent in PHOTAR patients (Table 5).

**Table 5:** Comparison of risk factors for young adult stroke between PHOTAR and PUFH patients

	PHOTAR (***n*** = 41)	PUFH (***n*** = 57)	***P***-Value
**Hypertension, n (%)**	15 (36.6)	40 (70.2)	0.002
**Diabetes mellitus, n (%)**	5 (12.2)	22 (47.8)	0.001
**Hyperlipidaemia, n (%)**	17 (41.5)	37 (64.9)	0.036
**Smoking history, n (%)**	16 (39.0)	30 (52.6)	0.260
**Alcohol history, n (%)**	16 (39.0)	32 (56.1)	0.142
**Stroke history, n (%)**	1 (2.4)	10 (17.5)	0.023
**Atrial fibrillation, n (%)**	0	0	None
**Erythrocytosis, n (%)**	12 (29.3)	0 (0)	< 0.001
**Hyperhomocysteinemia, n (%)**	19 (46.3)	22 (46.8)	1.000
**Severity of carotid artery atherosclerosis**			0.001
**Normal, n (%)**	29 (70.7)	16 (32,0)	
**Intima thickening, n (%)**	0 (0)	6 (12.0)	
**Plaque, n (%)**	8 (19.5)	25 (50.0)	
**Arteriostenosis, n (%)**	2 (4.9)	2 (4.0)	
**Arterial occlusion, n (%)**	2 (4.9)	1 (6.0)	

*PHOTAR* People’s Hospital of Tibet Autonomous Region, *PUFH* Peking University First Hospital

## Discussion

### Patient risk factors

The elevations of Beijing and Tibet are about 40 m and 3650 m above sea level, respectively. Two of the best hospitals in Beijing and Tibet, PUFH and PHOTAR, respectively, were chosen and represent top-level medical facilities. In PHOTAR, while the average age of AIS onset was about 4 years older compared to that of 10 years ago, it was still 7 years earlier than that of PUFH patients [6]. As suggested in the present study, this difference could be due to independent risk factors, erythrocytosis, and hyperhomocysteinemia, where the former could have resulted from high altitude and chronic anoxia and the latter from a large quantity diet of animal protein. Similarly, erythrocytosis was also a potential risk factor for high incidences of young adult stroke in PHOTAR patients, but since it was not detected in PUFH patients, accurate quantitative analysis was not conducted. Additionally, the fact that hypertension, DM, hyperlipidaemia, stroke history, and severity of carotid artery atherosclerosis were significantly less common in PHOTAR patients compared with those in PUFH patients could partially compensate for the difference due to the influence of erythrocytosis between two hospitals. Furthermore, the lower average age in Tibet could be explained by the speculation that younger patients in Tibet were more likely to seek medical care than older patients due to better financial capacity and education [12]. Therefore, future work with a well-controlled prospective cohort study would be required to better examine young adult stroke and the role of erythrocytosis in the Tibetan population. In both patient groups, the disproportionate presence of males was also higher than the average male-to-female ratio in their respective general population. This could be due to regional lifestyle and dietary habits since no significant sex-based differences have been described in both settings [4, 7, 13, 14].

### Intravenous thrombolysis

While 17.1% and 9.7% of patients arrived at PUFH and PHOTAR, respectively, within the IVTWT, only 5.2% and 0.4% of patients received IVT. Compared with PHOTAR patients, PUFH patients presented with a better realisation of AIS and a stronger desire to consult doctors. In fact, a study of 1.11 million hospitalized AIS cases in Germany has previously shown an IVT rate of 15.9% [15]. In addition to the lack of appropriate education related to AIS, low access to thrombolysis was primarily due to the economic burden that was more severe in Tibet, which is relatively underdeveloped. The thrombotic approaches used between the two hospitals also showed a latent difference. At PHOTAR, urokinase seemed to have a higher utilization rate due to a broader time window and a lower price. Because of a low number of patients with thrombolysis at PHOTAR, this difference should be considered carefully.

### Distribution of stroke subtypes and infarction location

Most cases of AIS are caused by thrombosis or thromboembolism. The TOAST classification and OCSP classification were established to better evaluate AIS based on its mechanism and location. The LAAS subtype of the TOAST classification was slightly more widespread in PHOTAR patients; atherosclerotic risk factors and carotid artery condition results in such patients were less severe than those in PUFH patients. Similarly, according to the OSCP classification, the TACI and PACI subtypes were significantly more prevalent in PHOTAR patients, while the LACI subtype was significantly more frequent in PUFH patients. Erythrocytosis might have contributed to the anterior circulation tendency in PHOTAR patients, as had been previously demonstrated in a series of studies [16–23]. Here, the underlying mechanism may be associated with viscosity and haematocrit, which do not follow a linear pattern, but rather have a theoretical “threshold effect” [21]. In this condition, a small increase above the haematocrit threshold may cause a huge change in viscosity and lead to further blood flow disturbance, finally resulting in ischemic events [19, 21, 24]. Because of the complex anterior cerebral circulation with a multistage branching system, anterior circulation cerebral infarction and watershed-distribution infarction may be noted more often [19, 20]. Any blood capacity variations may change the haematocrit and cause an ischemic stroke. Even though the vessel condition was less severe in carotid artery atherosclerosis, the LAAS subtype accounted for a comparably large proportion of AIS in PHOTAR patients. To establish an overall vessel condition profile, especially for intracranial arteries, more studies are required. Future studies should investigate if and how this process is influenced by the chronic structural and functional changes in cerebral tissue resulting from anoxia at high-altitude areas. It is also possible that high haematocrit is associated with an increased AIS risk. However, a prospective European study of 1638 polycythaemia patients with haematocrit of 0.472 ± 0.063 L/L has previously failed to show any relationship between increasing haematocrit and thrombotic events and has advocated for aggressive control of these parameters in patients with erythrocytosis [25]. Another retrospective study has suggested that elevated HGB on initial admission could be associated with more severe stroke, greater disability at discharge, and higher 30-day mortality rates after AIS, while lower HGB could indicate prolonged stay in the acute care hospital [18]. To improve AIS prevention, future studies should aim to establish the critical threshold value of HGB or haematocrit in patients with polycythaemia and elucidate the risks and underlying pathology of high HGB and haematocrit levels in stroke patients who are at a high altitude but do not meet the criteria for erythrocytosis.

### Treatment

During the hospitalisation phase, antiplatelet therapy was prescribed more commonly to PUFH patients than in PHOTAR patients. Indeed, the high rates of antihypertensive antidiabetic and antihyperlipidemic drug administration in PUFH patients reflected the high prevalence of these diseases. Differences in the use of ICP-lowering and anticoagulant drugs could be related to the different AIS pathogeneses. In PHOTAR patients, AIS was likely related to haemodynamic changes, affected a larger area and tended to cause oedema, while in the PUFH group, AIS was likely associated with atherosclerosis and thrombosis, where patients presented with more progressive stroke [26].

### Limitations

First, information regarding severity and prognosis, such as the National Institutes of Health Stroke Scale, the Modified Rankin Scale, or fatality of the disease was not assessed, and this may have resulted in an inadequate understanding of the two groups. Second, our study was based on hospitalized patients only. As some stroke patients treated in the emergency units may not have been admitted as inpatients, this may have contributed to AIS assessment bias. Third, even though both cohorts from the two hospitals contained more than 200 cases, the size of the cohorts was different, which may contribute to bias and thus, the results of this study should be interpreted carefully. Fourth, data on surgical operations such as thrombectomy were missing, which may result in an inadequate evaluation of the two groups. Finally, other factors such as treatment compliance, work intensity, emotional status, and sleep duration were not included in this study, which could also result in an inadequate evaluation of the two groups. Nevertheless, to our knowledge, this is the first hospital-based comparative study to describe AIS in Tibet.

## Conclusions

In Tibet, the average age of stroke patients was lower, and erythrocytosis and hyperhomocysteinemia were the independent risk factors of ischemic stroke. Young adult strokes and anterior circulation infarctions were more common in PHOTAR, possibly because of erythrocytosis induced by the high altitude. Future prospective cohort studies should aim to uncover the underlying pathology to improve AIS prevention further. Enhancing patient education in Tibet can contribute to better management of erythrocytosis and hyperhomocysteinemia and can potentially improve AIS prognosis.

## Data Availability

The datasets used and/or analysed during the current study are available from the corresponding author on reasonable request.

## Abbreviations

AIS: Acute ischemic stroke
PHOTAR: People’s Hospital of Tibet Autonomous Region
PUFH: Peking University First Hospital
IVTWT: Intravenous thrombolysis window time
TOAST: Trial of Org 10172 in Acute Stroke Treatment
OCSP: Oxfordshire Community Stroke Project classification
TACI: Total anterior circulation infarcts
PACI: Partial anterior circulation infarcts
